# Ovarian dysgerminoma in pregnancy: A case report

**DOI:** 10.1097/MD.0000000000025364

**Published:** 2021-04-09

**Authors:** Ioan Sas, Denis Mihai Şerban, Larisa - Cristina Tomescu, Nicoleta Nicolae

**Affiliations:** Department of Obstetrics and Gynecology, ”Victor Babeş“ University of Medicine and Pharmacy Timişoara, Romania, Eftimie Murgu Sq. no. 2, Timişoara, RO.

**Keywords:** dysgerminoma, germ cell malignancy, germ cells of the ovary, pregnancy

## Abstract

**Rationale:**

Although dysgerminomas are relatively uncommon among all ovarian neoplasms, representing for only about 2%, they account for 32.8 percent of malignant ovarian germ cell tumors. Their association with pregnancy is extremely rare; due to the low frequency of occurrence, there are few recommendations regarding pregnancy management; therefore, it is important to discuss and summarize the treatment strategy.

**Patient concerns:**

We present the case of a 25 years patient, gestation 1, para 1, who was hospitalized in the clinic at 38/39 weeks of gestation at the beginning of labor. Following the ultrasound examination, a hypoechogenic lesion on the uterine fundus was found, suggestive of subterranean fibroid. After caesarean section, right adnexectomy was performed; the histopathological examination revealed, unexpectedly, the diagnosis of dysgerminoma.

**Diagnoses:**

Dysgerminoma as associated with pregnancy.

**Interventions:**

Birth by Caesarean section and right adnexectomy. No other medical complications occurred.

**Outcomes:**

The histopathological and immunohistochemical examinations were consistent with the pure dysgerminoma. Oncology was staged AI, with the monitoring of markers and abdominal and pelvic magnetic resonance imaging at 3, 6, 9, and 12 months.

**Lessons:**

Dysgerminoma is the most common ovarian malignancy associated with pregnancy with a good fetal maternal outcome. If these tumors are discovered accidentally during caesarean section, tumor markers and magnetic resonance imaging scanning should be done postoperatively to plan optimal treatment.

## 1 Introduction

Malignant germ cell tumor is a relatively uncommon subtype of ovarian cancer accounting for less than 5% of all ovarian cancers. The incidence of any type of ovarian cancer is 2.8 to 11 per 100,000 pregnancies.^[1]^ Although dysgerminomas are relatively uncommon among all ovarian neoplasms (accounting for only an approximate 2 percent), they account for 32.8 percent of malignant ovarian germ cell tumors.^[2]^ The majority of cases (75 percent) arise in adolescents and young adults, in whom they account for approximately one-third of all ovarian malignant neoplasms.^[3]^ Because of their predilection for young women, they are 1 of the more common ovarian malignant neoplasms detected during pregnancy. In other words, the incidence of ovarian dysgerminoma is about 0.2 to 1 per 1000000 pregnancies; therefore, ovarian dysgerminoma in pregnancy is an extremely rare clinical condition. Its management remains a challenge, in the specialized literature only sporadic cases have been reported. The purpose is to report and discuss our case, as further studies are needed to confirm the best management especially in the management of the incidentally diagnosed tumor.

## 2 Case report

Patient 25 years old, gestation 1, para 1, was hospitalized to our clinic at 38/39 weeks of gestation at the beginning of labor, without a history of heredocolateral or personal pathology.

At the abdominal examination, the height of the uterine bottom was 38  centimeter , and the abdominal circumference was 100  centimeter , with cephalic presentation. A firm swelling was felt adjacent to the uterus on the right side, with reduced mobility. Her laboratory tests after admission revealed mild anemia (hemoglobin 10.59 g/dL), increased liver function tests: aspartate aminotransferase 80 U/L (from 8 to 37 U/L) and alanin aminotransferase 102 U/L (from 8 to 65 U/L).

At the ultrasound examination we found a pregnancy with a single fetus, alive for 38 weeks and 6 days with cephalic presentation. The placenta was located on the posterior wall in the upper uterine segment. Amniotic fluid in normal quantity. Doppler measurements on: umbilical artery pulsatility index = 0.82, mean cerebral artery pulsatility index = 1.40 and Heart rate = 143b / min. A hypoechogenic lesion was observed on the right uterus approximately 12x11  centimeter, which was reported as a subsoil fibroma.

**Figure 1:**
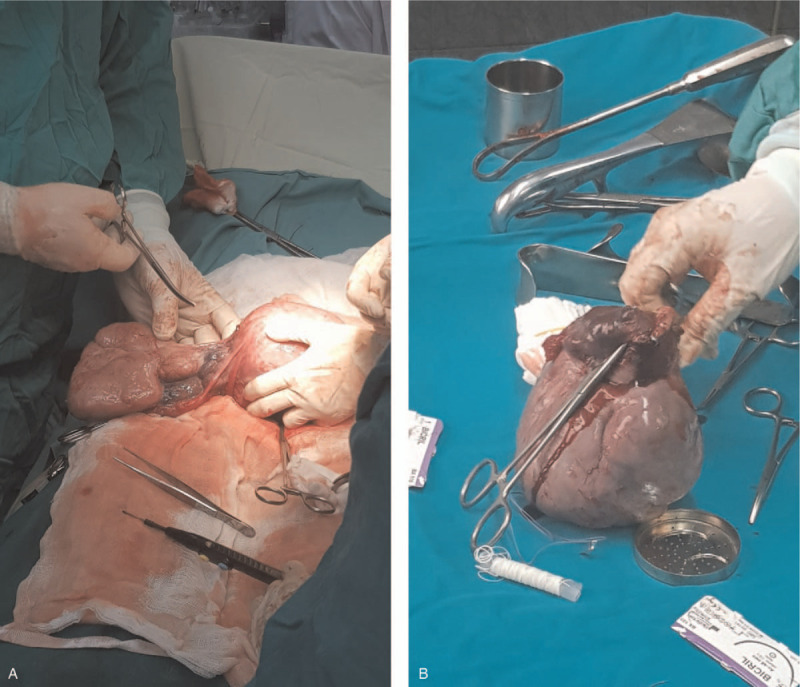
A - The right ovarian mass and Fallopian tube before excision. B - The right ovarian mass and Fallopian tube after excision.

**Figure 2:**
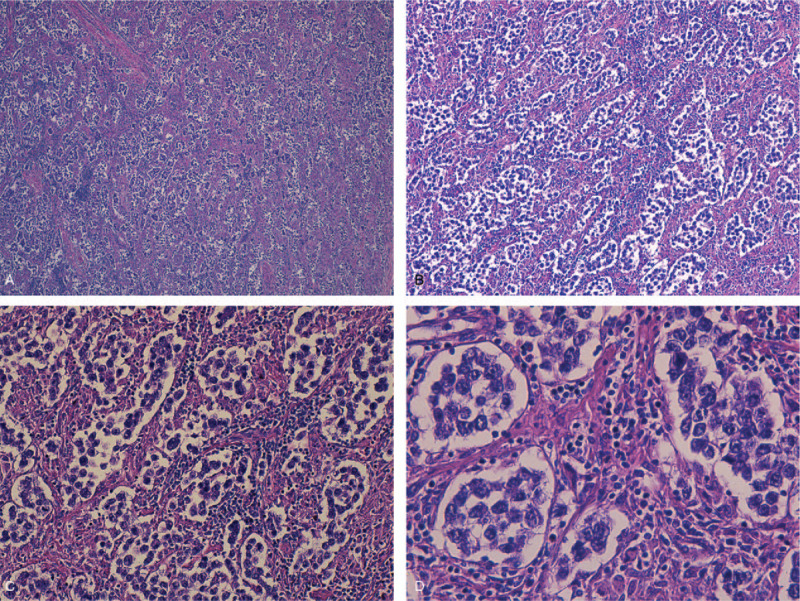
A - Histopathological results of the ovarian dysgerminoma and immunohistochemical staining H&E stain, x4, tumor proliferation with varied architectural pattern, tumor cells arranged in cords, trabeculae, nests or forming pseudovascular spaces, tumor stroma generally reduced, represented by fibrous spines and numerous lymphocytes. B - Histopathological results of the ovarian dysgerminoma and immunohistochemical staining H&E stain, x10, tumor proliferation with varied architectural pattern, tumor cells arranged in cords, trabeculae, nests or forming pseudovascular spaces, tumor stroma generally reduced, represented by fibrous spines and numerous lymphocytes. C - Histopathological results of the ovarian dysgerminoma and immunohistochemical staining H&E stain, x20, tumor proliferation with varied architectural pattern, tumor cells arranged in cords, trabeculae, nests or forming pseudovascular spaces, tumor stroma generally reduced, represented by fibrous spines and numerous lymphocytes. D - Histopathological results of the ovarian dysgerminoma and immunohistochemical staining H&E stain, x40, tumor proliferation with varied architectural pattern, tumor cells arranged in cords, trabeculae, nests or forming pseudovascular spaces, tumor stroma generally reduced, represented by fibrous spines and numerous lymphocytes.

**Figure 3:**
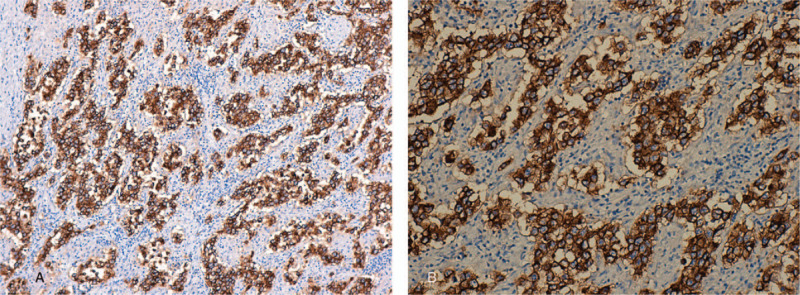
A - Histopathological results of the ovarian dysgerminoma and immunohistochemical staining Immunohistochemical reactions: Tumor cells were positive for: CD117x10. B - Histopathological results of the ovarian dysgerminoma and immunohistochemical staining Immunohistochemical reactions: Tumor cells were positive for: CD117x20. CD117 = monoclonal antibody targeting c-Kit.

**Figure 4:**
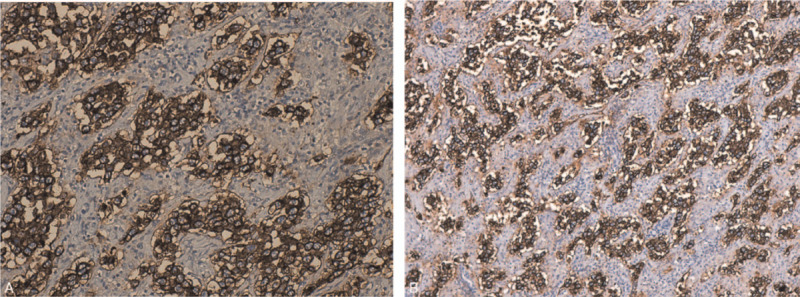
A - Histopathological results of the ovarian dysgerminoma and immunohistochemical staining Immunohistochemical reactions: Tumor cells were positive for: D2-40 X10. B - Histopathological results of the ovarian dysgerminoma and immunohistochemical staining Immunohistochemical reactions: Tumor cells were positive for: D2-40 X20. D2-40 = podoplanin.

**Figure 5:**
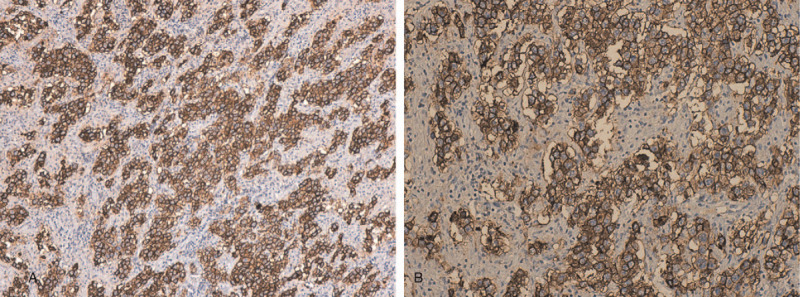
A - Histopathological results of the ovarian dysgerminoma and immunohistochemical staining Immunohistochemical reactions: Tumor cells were positive for: PLAP x10. B - Histopathological results of the ovarian dysgerminoma and immunohistochemical staining Immunohistochemical reactions: Tumor cells were positive for: PLAP x10. PLAP = placental alkaline phosphate.

The decision to end the pregnancy was made taking into account the term pregnancy and the beginning of labor. She gave birth to a live, healthy, 3900 g female fetus, with the Apgar Index = 9 by Caesarean section, with the negative labor sample indication. Intraoperatively, on the right side a firm ovarian mass was observed, with the capsule intact (Fig. 1A, Fig. 1B)); the right fallopian tube extended and adherent over the tumor mass without identifying healthy ovarian tissue on the right ovary. No anomalies were found in the left appendix. No ascites fluid. Right adnexectomy was performed. No enlarged para-aortic or retroperitoneal lymph nodes were observed; all other structures in the abdomen and pelvis were normal. On the cut section, the tumor mass was homogeneous, gray-white, without cystic or hemorrhagic changes. The intra- and postoperative evolution was favorable, with the normalization of liver function tests and mild anemia (hemoglobin 9.3 g/dl). Histology unexpectedly revealed the diagnosis of dysgerminoma, tumor proliferation with varied architectural pattern, tumor cells arranged in cords, trabeculae, nests or forming pseudo-vascular spaces, tumor stroma generally reduced, represented by fibrous spines and numerous lymphocytes (Fig. 2A, Fig. 2B, Fig. 2C, Fig. 2D). Immunohistochemical staining markers OCT3 / 4, monoclonal antibody targeting c-Kit (Figure 3A, Fig. 3B), podoplanin (Fig. 4A, Fig. 4B) and placental alkaline phosphate (Fig. 5A, Fig. 5B) were positive, and for alpha fetoprotein, and negative panCK. Postoperatively, no abnormal ultrasound and magnetic resonance imaging findings were detected. Serum cancer antigen 125, lactic dehydrogenase (LDH) and total human chorionic gonadotropin = 1.0 mIU / mL (normal value = <5.3 mIU / ml) were also within normal limits. The patient was reassessed at 3, 6, 9, and 12 months with markers within normal limits and normal abdominal and pelvic magnetic resonance imaging.

## 3 Discussion

Ovarian Germ Cell Tumors comprise 27,1% of all ovarian cancers.^[4]^ Only 3% are malignant, dysgerminomas, being the most common. only 3% are malign, dysgerminomas being the most frequent. This case needs reporting and discussion, because its management remains a challenge with only sporadic cases.

These tumors may be asymptomatic or can present with pain in abdomen, misdiagnosed as acute appendicitis, ectopic pregnancy, acute abdomen, distension of abdomen with vaginal bleeding.^[5]^ These tumors can affect conception and if pregnancy occurs, it can lead to maternal-fetal compromise. Increased risk of torsion, incarceration, rupture and hemorrhage can occur during pregnancy and vaginal delivery. Fetal demise has been reported to be in 25% of cases.^[6]^ In our case the tumor was asymptomatic, without maternal-fetal involvement.

Generally, the main differential diagnosis of dysgerminoma is lymphoma. But macroscopic and microscopic features of dysgerminoma can be confused with lymphoma. Other differential diagnoses include solid type of yolk sac tumor, embryonal carcinoma and Sertoli cell tumor which were all excluded by morphology and immunohistochemistry.^[7]^

Dysgerminomas can contain syncytiotrophoblastic giant cells that produce placental alkaline phosphatase, and LDH.^[8,9]^ Serial measurements of these markers can be useful for monitoring disease. In addition, 3 -5% of dysgerminomas produce human chorionic gonadotropin. In general, dysgerminomas do not produce alpha fetoprotein, although borderline elevations (<16 ng/mL) are described in case series, but most often in the setting of mixed germ cell tumors that contain a yolk sac element.

75% of women with dysgerminomas present with stage I disease; the contralateral ovary is involved in 10% to 15%.^[10,11]^ Bilateral ovarian disease is more common with dysgerminoma than with any other malignant OGCT.

Surgery is performed for definitive diagnosis, staging, and initial treatment. For a unilateral neoplasm confined to the ovary without capsular involvement or rupture, simple salpingo-oophorectomy is curative in over 95 percent.

The management of ovarian cancer in pregnancy is complicated, as there are 3 separate but interactive parts, that is, mother, fetus, and malignancy, which must be managed simultaneously. Therefore, the decisions regarding each case should be on an individual basis, taking into consideration the patient's age, parity, desire for present pregnancy, future fertility, stage of the tumor, and duration of gestation.

If pregnancy is desired, in general, abdominal surgery should be undertaken in the second trimester because the risk of miscarriage is decreased and the size of uterus still allows a certain degree of access.^[12,13]^ First, according to committee opinion of the American Society of Anesthesiologists, no currently used anesthetic agents have been shown to have any teratogenic effects in humans when using standard concentrations at any gestational age.^[13]^

If pregnancy is desired, ideal time for intervention is 16 to 18 weeks of gestation with removal of tumor and surgical staging. Chemotherapy can be given after surgery, though feto-maternal complications that is, abortions, haemopoietic depression, infections, fetal pancytopenia, delayed cognitive development, and low birth weight can occur.^[14]^

In stage 1A, conservative or fertility sparing surgery can be done if patient is desirous of pregnancy and includes- staging laparotomy with unilateral salpingo-oophrectomy. No chemotherapy is required for stage 1A tumors unless recurrence^[15]^ (9.2% cases) occurs. Treatment for stage 1B is bilateral salpingo-oophrectomy with or without total abdominal hysterectomy^[16]^ followed by 3 cycles of Bleomycin, etoposide and platinum chemotherapy^[17]^ as was done in our case. However, fertility sparing surgery can be done even in bilateral dysgerminomas if patient is desirous of future pregnancy as no difference in outcome between fertility sparing and non-conservative surgery has been found.^[15]^ Treatment for stage II, III & IV is complete resection followed by 4 cycles of Bleomycin, etoposide, and platinum chemotherapy.^[17]^ Patients with bulky residual disease require additional cycles.

Though the recurrence rates have been reported more in the incompletely staged or unstaged patients,^[18]^ yet these tumors are highly chemosensitive and prognosis is excellent. Follow-up should be done every 3-4 monthly for 3 years, then 6 monthly for next 2 years and then yearly up to 10 years. computerized tomography scan should be done at 6, 12 months especially if markers are negative at diagnosis. Follow- up with serum LDH can be done. The materno-fetal outcome of patients with pregnancy with dysgerminoma is excellent. A 97% to 100% survival rate with ovarian dysgerminoma has been reported.^[19]^

## 4 Conclusion

Pregnant women with dysgerminoma are young and have good feto-maternal outcome. Fertility sparing surgery can be offered in women, desirous of pregnancy. The treatment strategy must be discussed and structured individually

If these tumors are found incidentally during caesarean section, tumor markers and computerized tomography scan should be done postoperatively.

## Acknowledgments

We extend our gratitude to the team in the Gynecology and Obstetrics unit as well as to the pathologist and oncologist for their support and interdisciplinary approach.

## Author contributions

**Conceptualization:** Ioan Sas, Larisa - Cristina Tomescu.

**Investigation:** Ioan Sas.

**Writing – original draft:** Ioan Sas, Larisa - Cristina Tomescu.

**Writing – review & editing:** Denis Mihai Şerban, Nicoleta Nicolae.
